# New records of Trichoceridae (Diptera) from the island of Mallorca ​

**DOI:** 10.3897/BDJ.4.e7610

**Published:** 2016-01-21

**Authors:** Andrius Petrašiūnas, Gunnar Mikalsen Kvifte

**Affiliations:** ‡ Vilnius university, Vilnius, Lithuania; § University Museum of Bergen, Bergen, Norway

**Keywords:** Trichoceridae, faunistics, new records, Mallorca, Majorca, Balearic Islands, winter gnats

## Abstract

**Background:**

The Trichoceridae are a small family distributed mainly in the Holarctic Region, most of which are associated with cold seasons and even snow. From the Iberian peninsula, 5 species have been recorded; however only a single previous occurence record exists from the Balearic islands.

**New information:**

In this paper we present new records of two species from Mallorca, of which Trichocera (Saltrichocera) saltator (Harris, 1776) has not previously been recorded from the Balearic islands. Trichocera (Saltrichocera) annulata Meigen, 1818 is recorded for the first time from Mallorca. We furthermore discuss the species' distributions within the Mediterranean region and report new morphological data for the Mallorca island form of *T. saltator*.

## Introduction

Winter gnats (Diptera: Trichoceridae) are a small family that includes approximately 160 recent species, 110 of which are classified in the widespread genus *Trichocera* Meigen, 1803. Species of this genus are mainly distributed in the Holarctic region and prefer lower temperatures, so are mostly observed during the colder season and even on snow.

In the catalogue of Diptera of Spain, Portugal and Andorra, Dahl et al. (2002) listed 5 species of Trichoceridae known from Spain with two of those – Trichocera (Saltrichocera) annulata Meigen, 1818 and Trichocera (Saltrichocera) maculipennis Meigen, 1818 – recorded from the Canary islands. There were no records of Trichoceridae from Balearic islands until 2009, when one male of Trichocera (S.) annulata was found on Menorca island (Carles-Tolrá and Ventura 2009).

Here we present further data on winter gnats of Balearic islands, with new records of this family from the island of Mallorca.

## Materials and methods

**Figure 1.**
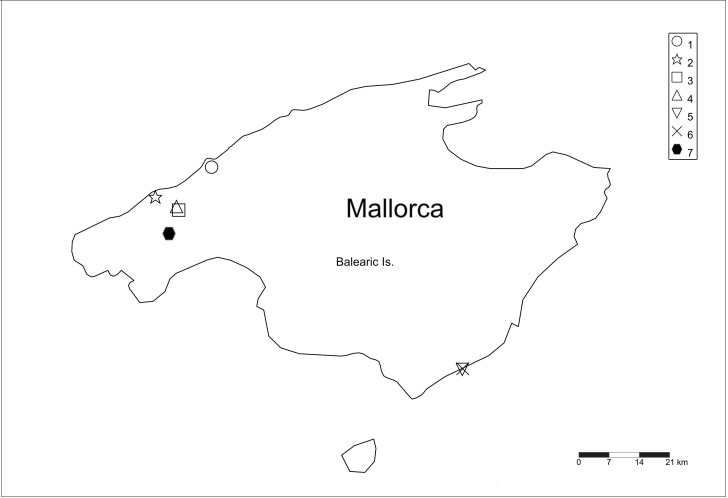
Map of localities for Mallorcan *Trichocera* (see Table 1 for locality details).

Specimens were collected by sweeping at various localities in Mallorca during February 2015, and preserved in 70–100% alcohol. Voucher specimens are retained in Museum of Zoology of Vilnius University (MZVU), Vilnius, Lithuania. Localities visited are given in Fig. 1 and the map was produced using the SimpleMappr online tool (Shorthouse 2010). Temperatures given in Table 1 are taken from the closest meterological measuring stations presented by Meteo de Mallorca (2016); and are given as the minimum and the maximum measured temperature on the day of sampling.

## Taxon treatments

### Trichocera (Saltrichocera) annulata

Meigen, 1818

#### Materials

- **Type status:** Other material. **Occurrence:** individualCount: 1; sex: male; lifeStage: adult; **Taxon:** scientificNameID: Trichocera (Saltrichocera) annulata Meigen, 1818; scientificName: Trichocera (Saltrichocera) annulata Meigen, 1818; kingdom: Animalia; phylum: Arthropoda; class: Hexapoda; order: Diptera; family: Trichoceridae; genus: Trichocera; subgenus: Saltrichocera; specificEpithet: annulata; scientificNameAuthorship: Meigen, 1818; **Location:** country: Spain; stateProvince: Balearic Islands; county: Mallorca; locality: Esporles, Son Tria recreational area; verbatimLocality: Spain, Mallorca, Esporles, Son Tria recreational area; verbatimLatitude: 39.663051; verbatimLongitude: 2.573698; decimalLatitude: 39.663051; decimalLongitude: 2.573698; georeferenceProtocol: label; georeferenceSources: Google Maps; **Identification:** identifiedBy: Andrius Petrašiūnas; dateIdentified: 2015; **Event:** samplingProtocol: sweep net; year: 2015; month: feb; day: 9; habitat: streambank and fountain in recreational area; **Record Level:** basisOfRecord: PreservedSpecimen
- **Type status:** Other material. **Occurrence:** individualCount: 2; sex: male; lifeStage: adult; **Taxon:** scientificNameID: Trichocera (Saltrichocera) annulata Meigen, 1818; scientificName: Trichocera (Saltrichocera) annulata Meigen, 1818; kingdom: Animalia; phylum: Arthropoda; class: Hexapoda; order: Diptera; family: Trichoceridae; genus: Trichocera; subgenus: Saltrichocera; specificEpithet: annulata; scientificNameAuthorship: Meigen, 1818; **Location:** country: Spain; stateProvince: Balearic Islands; county: Mallorca; locality: Esporles, Torrent de Son Vic; verbatimLocality: Spain, Mallorca, Esporles, Torrent de Son Vic; verbatimLatitude: 39.670459; verbatimLongitude: 2.569193; decimalLatitude: 39.670459; decimalLongitude: 2.569193; georeferenceProtocol: label; georeferenceSources: Google Maps; **Identification:** identifiedBy: Andrius Petrašiūnas; dateIdentified: 2015; **Event:** samplingProtocol: sweep net; year: 2015; month: feb; day: 10; habitat: vegetation on stream banks; **Record Level:** basisOfRecord: PreservedSpecimen
- **Type status:** Other material. **Occurrence:** individualCount: 1; sex: female; lifeStage: adult; **Taxon:** scientificNameID: Trichocera (Saltrichocera) annulata Meigen, 1818; scientificName: Trichocera (Saltrichocera) annulata Meigen, 1818; kingdom: Animalia; phylum: Arthropoda; class: Hexapoda; order: Diptera; family: Trichoceridae; genus: Trichocera; subgenus: Saltrichocera; specificEpithet: annulata; scientificNameAuthorship: Meigen, 1818; **Location:** country: Spain; stateProvince: Balearic Islands; county: Mallorca; locality: Esporles, Torrent de Son Vic; verbatimLocality: Spain, Mallorca, Esporles, Torrent de Son Vic; verbatimLatitude: 39.670459; verbatimLongitude: 2.569193; decimalLatitude: 39.670459; decimalLongitude: 2.569193; georeferenceProtocol: label; georeferenceSources: Google Maps; **Identification:** identifiedBy: Andrius Petrašiūnas; dateIdentified: 2015; **Event:** samplingProtocol: sweep net; year: 2015; month: feb; day: 10; habitat: vegetation on stream banks; **Record Level:** basisOfRecord: PreservedSpecimen
- **Type status:** Other material. **Occurrence:** individualCount: 2; sex: male; lifeStage: adult; **Taxon:** scientificNameID: Trichocera (Saltrichocera) annulata Meigen, 1818; scientificName: Trichocera (Saltrichocera) annulata Meigen, 1818; kingdom: Animalia; phylum: Arthropoda; class: Hexapoda; order: Diptera; family: Trichoceridae; genus: Trichocera; subgenus: Saltrichocera; specificEpithet: annulata; scientificNameAuthorship: Meigen, 1818; **Location:** country: Spain; stateProvince: Balearic Islands; county: Mallorca; locality: Banyalbufera; verbatimLocality: Spain, Mallorca, Banyalbufera; verbatimLatitude: 39.690612; verbatimLongitude: 2.525107; decimalLatitude: 39.690612; decimalLongitude: 2.525107; georeferenceProtocol: label; georeferenceSources: Google Maps; **Identification:** identifiedBy: Andrius Petrašiūnas; dateIdentified: 2015; **Event:** samplingProtocol: sweep net; year: 2015; month: feb; day: 9; habitat: seashore cliffs; **Record Level:** basisOfRecord: PreservedSpecimen
- **Type status:** Other material. **Occurrence:** individualCount: 1; sex: male; lifeStage: adult; **Taxon:** scientificNameID: Trichocera (Saltrichocera) annulata Meigen, 1818; scientificName: Trichocera (Saltrichocera) annulata Meigen, 1818; kingdom: Animalia; phylum: Arthropoda; class: Hexapoda; order: Diptera; family: Trichoceridae; genus: Trichocera; subgenus: Saltrichocera; specificEpithet: annulata; scientificNameAuthorship: Meigen, 1818; **Location:** country: Spain; stateProvince: Balearic Islands; county: Mallorca; locality: Deiá, Tributary to Torrent Major; verbatimLocality: Spain, Mallorca, Deiá, Tributary to Torrent Major; verbatimLatitude: 39.752634; verbatimLongitude: 2.642415; decimalLatitude: 39.752634; decimalLongitude: 2.642415; georeferenceProtocol: label; georeferenceSources: Google Maps; **Identification:** identifiedBy: Andrius Petrašiūnas; dateIdentified: 2015; **Event:** samplingProtocol: sweep net; year: 2015; month: feb; day: 8; habitat: rocky stream bed with bryophytes; **Record Level:** basisOfRecord: PreservedSpecimen
- **Type status:** Other material. **Occurrence:** individualCount: 1; sex: male; lifeStage: adult; **Taxon:** scientificNameID: Trichocera (Saltrichocera) annulata Meigen, 1818; scientificName: Trichocera (Saltrichocera) annulata Meigen, 1818; kingdom: Animalia; phylum: Arthropoda; class: Hexapoda; order: Diptera; family: Trichoceridae; genus: Trichocera; subgenus: Saltrichocera; specificEpithet: annulata; scientificNameAuthorship: Meigen, 1818; **Location:** country: Spain; stateProvince: Balearic Islands; county: Mallorca; locality: Cala Figuera; verbatimLocality: Spain, Mallorca, Cala Figuera; verbatimLatitude: 39.330871; verbatimLongitude: 3.165476; decimalLatitude: 39.330871; decimalLongitude: 3.165476; georeferenceProtocol: label; georeferenceSources: Google Maps; **Identification:** identifiedBy: Andrius Petrašiūnas; dateIdentified: 2015; **Event:** samplingProtocol: sweep net; year: 2015; month: feb; day: 11; habitat: open forest glade in town; **Record Level:** basisOfRecord: PreservedSpecimen
- **Type status:** Other material. **Occurrence:** individualCount: 2; sex: male; lifeStage: adult; **Taxon:** scientificNameID: Trichocera (Saltrichocera) annulata Meigen, 1818; scientificName: Trichocera (Saltrichocera) annulata Meigen, 1818; kingdom: Animalia; phylum: Arthropoda; class: Hexapoda; order: Diptera; family: Trichoceridae; genus: Trichocera; subgenus: Saltrichocera; specificEpithet: annulata; scientificNameAuthorship: Meigen, 1818; **Location:** country: Spain; stateProvince: Balearic Islands; county: Mallorca; locality: Cala Figuera; verbatimLocality: Spain, Mallorca, Cala Figuera; verbatimLatitude: 39.332245; verbatimLongitude: 3.166440; decimalLatitude: 39.332245; decimalLongitude: 3.166440; georeferenceProtocol: label; georeferenceSources: Google Maps; **Identification:** identifiedBy: Andrius Petrašiūnas; dateIdentified: 2015; **Event:** samplingProtocol: sweep net; year: 2015; month: feb; day: 11; habitat: vegetation in canyon near port; **Record Level:** basisOfRecord: PreservedSpecimen
- **Type status:** Other material. **Occurrence:** individualCount: 3; sex: female; lifeStage: adult; **Taxon:** scientificNameID: Trichocera (Saltrichocera) annulata Meigen, 1818; scientificName: Trichocera (Saltrichocera) annulata Meigen, 1818; kingdom: Animalia; phylum: Arthropoda; class: Hexapoda; order: Diptera; family: Trichoceridae; genus: Trichocera; subgenus: Saltrichocera; specificEpithet: annulata; scientificNameAuthorship: Meigen, 1818; **Location:** country: Spain; stateProvince: Balearic Islands; county: Mallorca; locality: Cala Figuera; verbatimLocality: Spain, Mallorca, Cala Figuera; verbatimLatitude: 39.332245; verbatimLongitude: 3.166440; decimalLatitude: 39.332245; decimalLongitude: 3.166440; georeferenceProtocol: label; georeferenceSources: Google Maps; **Identification:** identifiedBy: Andrius Petrašiūnas; dateIdentified: 2015; **Event:** samplingProtocol: sweep net; year: 2015; month: feb; day: 11; habitat: vegetation in canyon near port; **Record Level:** basisOfRecord: PreservedSpecimen
- **Type status:** Other material. **Occurrence:** individualCount: 4; sex: male; lifeStage: adult; **Taxon:** scientificNameID: Trichocera (Saltrichocera) annulata Meigen, 1818; scientificName: Trichocera (Saltrichocera) annulata Meigen, 1818; kingdom: Animalia; phylum: Arthropoda; class: Hexapoda; order: Diptera; family: Trichoceridae; genus: Trichocera; subgenus: Saltrichocera; specificEpithet: annulata; scientificNameAuthorship: Meigen, 1818; **Location:** country: Spain; stateProvince: Balearic Islands; county: Mallorca; locality: Son Serralta; verbatimLocality: Spain, Mallorca, Son Serralta; verbatimLatitude: 39.614133; verbatimLongitude: 2.553593; decimalLatitude: 39.614133; decimalLongitude: 2.553593; georeferenceProtocol: label; georeferenceSources: Google Maps; **Identification:** identifiedBy: Andrius Petrašiūnas; dateIdentified: 2015; **Event:** samplingProtocol: sweep net; year: 2015; month: feb; day: 12; habitat: stream bank near bridge and waterfall; **Record Level:** basisOfRecord: PreservedSpecimen
- **Type status:** Other material. **Occurrence:** individualCount: 4; sex: female; lifeStage: adult; **Taxon:** scientificNameID: Trichocera (Saltrichocera) annulata Meigen, 1818; scientificName: Trichocera (Saltrichocera) annulata Meigen, 1818; kingdom: Animalia; phylum: Arthropoda; class: Hexapoda; order: Diptera; family: Trichoceridae; genus: Trichocera; subgenus: Saltrichocera; specificEpithet: annulata; scientificNameAuthorship: Meigen, 1818; **Location:** country: Spain; stateProvince: Balearic Islands; county: Mallorca; locality: Son Serralta; verbatimLocality: Spain, Mallorca, Son Serralta; verbatimLatitude: 39.614133; verbatimLongitude: 2.553593; decimalLatitude: 39.614133; decimalLongitude: 2.553593; georeferenceProtocol: label; georeferenceSources: Google Maps; **Identification:** identifiedBy: Andrius Petrašiūnas; dateIdentified: 2015; **Event:** samplingProtocol: sweep net; year: 2015; month: feb; day: 12; habitat: stream bank near bridge and waterfall; **Record Level:** basisOfRecord: PreservedSpecimen

#### Notes

**Figure 2.**
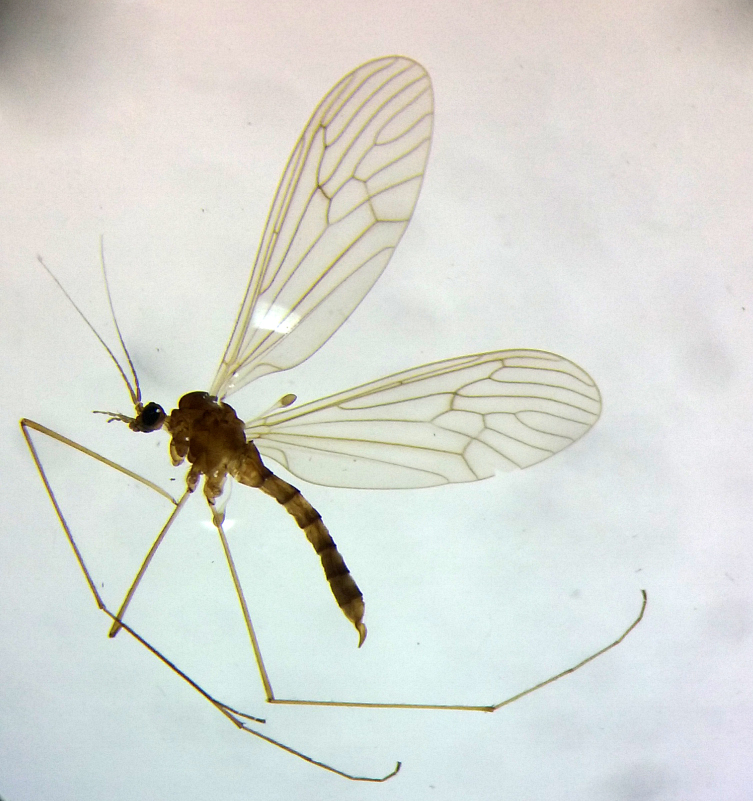
*Trichocera annulata* Meigen, 1818 female from Mallorca.

First records for Mallorca and second from the Balearic Islands (Fig. 2).

*Trichocera annulata* is widespread in the Mediterranean region, with records from Algeria (Edwards 1923), Asia Minor and Ethiopia (Dahl and Alexander 1976), Malta (Ebejer 2015), Morocco (Driauach et al. 2015) and Sardinia (Petrašiūnas 2009). Along with our data from Mallorca, these records suggests that this species has an unusually high tolerance to warm temperatures within the genus *Trichocera*.

### Trichocera (Saltrichocera) saltator

(Harris, 1776)

#### Materials

- **Type status:** Other material. **Occurrence:** individualCount: 1; sex: male; lifeStage: adult; **Taxon:** scientificNameID: Trichocera (Saltrichocera) saltator (Harris, 1776); scientificName: Trichocera (Saltrichocera) saltator (Harris, 1776); kingdom: Animalia; phylum: Arthropoda; class: Hexapoda; order: Diptera; family: Trichoceridae; genus: Trichocera; subgenus: Saltrichocera; specificEpithet: saltator; scientificNameAuthorship: (Harris, 1776); **Location:** country: Spain; stateProvince: Balearic Islands; county: Mallorca; locality: Esporles, Torrent de Son Vic; verbatimLocality: Spain, Mallorca, Esporles, Torrent de Son Vic; verbatimLatitude: 39.670459; verbatimLongitude: 2.569193; decimalLatitude: 39.670459; decimalLongitude: 2.569193; georeferenceProtocol: label; georeferenceSources: Google Maps; **Identification:** identifiedBy: Andrius Petrašiūnas; dateIdentified: 2015; **Event:** samplingProtocol: sweep net; year: 2015; month: feb; day: 10; habitat: vegetation on stream banks; **Record Level:** basisOfRecord: PreservedSpecimen
- **Type status:** Other material. **Occurrence:** individualCount: 1; sex: male; lifeStage: adult; **Taxon:** scientificNameID: Trichocera (Saltrichocera) saltator (Harris, 1776); scientificName: Trichocera (Saltrichocera) saltator (Harris, 1776); kingdom: Animalia; phylum: Arthropoda; class: Hexapoda; order: Diptera; family: Trichoceridae; genus: Trichocera; subgenus: Saltrichocera; specificEpithet: saltator; scientificNameAuthorship: (Harris, 1776); **Location:** country: Spain; stateProvince: Balearic Islands; county: Mallorca; locality: Son Serralta; verbatimLocality: Spain, Mallorca, Son Serralta; verbatimLatitude: 39.614133; verbatimLongitude: 2.553593; decimalLatitude: 39.614133; decimalLongitude: 2.553593; georeferenceProtocol: label; georeferenceSources: Google Maps; **Identification:** identifiedBy: Andrius Petrašiūnas; dateIdentified: 2015; **Event:** samplingProtocol: sweep net; year: 2015; month: feb; day: 12; habitat: stream bank near bridge and waterfall; **Record Level:** basisOfRecord: PreservedSpecimen

#### Notes

Previous records from the Iberian peninsula are only known from the Biscaya province in Northern Spain (Carles-Tolrá and Saloña 2004).

**Figure 3.**
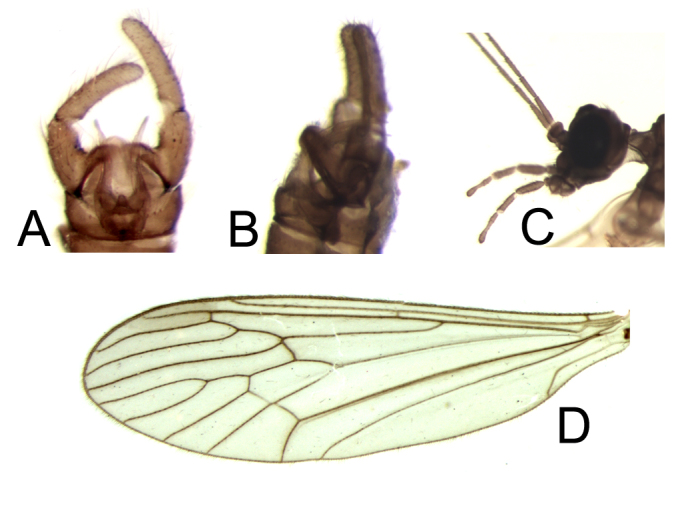
*Trichocera saltator* (Harris, 1766), morphological characters. A, Male genitalia, ventral view; B, Male genitalia, lateral view; C, Head with basal antennal segments, lateral view; D, Wing

Specimens of *T. saltator* from Mallorca have around 30 setae on the ventral side of Sc vein, which is a case similarly observed in males of *T. pappi* Krzeminska, 2003 from Morocco (Driauach et al. 2015). However, in other characters the Mallorcan specimens agree with *T. saltator*, including wing venation not typical for *T. pappi* and the species-specific male gonocoxal bridge and aedeagal complex (Fig. 3 A, B, D).

## Supplementary Material

XML Treatment for Trichocera (Saltrichocera) annulata

XML Treatment for Trichocera (Saltrichocera) saltator

## Figures and Tables

**Table 1.** Localities of Mallorcan Trichoceridae

**Locality number**	**North**	**East**	**Locality name**	**Habitat**	**Sampling date**	**Temperature range in centigrades**	**Collectors**
1	39.670459	2.569193	Esporles, Torrent de Son Vic	vegetation on stream banks	10.02.2015	3–15	G.Kvifte
2	39.752634	2.642415	Deia, Tributary to Torrent Major	rocky stream bed with bryophytes	08.02.2015	3–10	G.Kvifte, M. Stokkan & C.Garcia
3	39.663051	2.573698	Esporles, Son Tria recreational area	Vegetation near stream bank and fountain	09.02.2015	3–13	G.Kvifte & C.Garcia
4	39.690612	2.525107	Banyalbufera	Seaside cliffs	09.02.2015	3–13	G.Kvifte
5	39.332245	3.166440	Cala Figuera	vegetation in canyon near port	11.02.2015	5–16	G.Kvifte
6	39.330871	3.165476	Cala Figuera	open forest glade in town	11.02.2015	5–16	G.Kvifte
7	39.614133	2.553593	Son Serralta	stream bank near bridge and waterfall	12.02.2015	5–13	G.Kvifte
